# Cryptochrome Genes Are Highly Expressed in the Ovary of the African Clawed Frog, *Xenopus tropicalis*

**DOI:** 10.1371/journal.pone.0009273

**Published:** 2010-02-17

**Authors:** Yoko Kubo, Takahiro Takeuchi, Keiko Okano, Toshiyuki Okano

**Affiliations:** 1 Department of Electrical Engineering and Bioscience, Graduate School of Advanced Science and Engineering, Waseda University, Tokyo, Japan; 2 Precursory Research for Embryonic Science and Technology, Japan Science and Technology Agency, Tokyo, Japan; Vanderbilt University, United States of America

## Abstract

Cryptochromes (CRYs) are flavoproteins sharing high homology with photolyases. Some of them have function(s) including transcription regulation in the circadian clock oscillation, blue-light photoreception for resetting the clock phase, and light-dependent magnetoreception. Vertebrates retain multiple sets of CRY or CRY-related genes, but their functions are yet unclear especially in the lower vertebrates. Although CRYs and the other circadian clock components have been extensively studied in the higher vertebrates such as mice, only a few model species have been studied in the lower vertebrates. In this study, we identified two CRYs, XtCRY1 and XtCRY2 in *Xenopus tropicalis*, an excellent experimental model species. Examination of tissue specificity of their mRNA expression by real-time PCR analysis revealed that both the XtCRYs showed extremely high mRNA expression levels in the ovary. The mRNA levels in the ovary were about 28-fold (*XtCry1*) and 48-fold (*XtCry2*) higher than levels in the next abundant tissues, the retina and kidney, respectively. For the functional analysis of the XtCRYs, we cloned circadian positive regulator XtCLOCK and XtBMAL1, and found circadian enhancer E-box in the upstream of *XtPer1* gene. XtCLOCK and XtBMAL1 exhibited strong transactivation from the *XtPer1* E-box element, and both the XtCRYs inhibited the XtCLOCK:XtBMAL1-mediated transactivation, thereby suggesting this element to drive the circadian transcription. These results revealed a conserved main feedback loop in the *X. tropicalis* circadian clockwork and imply a possible physiological importance of CRYs in the ovarian functions such as synthesis of steroid hormones and/or control of estrus cycles via the transcription regulation.

## Introduction

The circadian clock uses properties of both self-sustained oscillation and sensitivity to environmental light for its resetting. In the molecular clock, several clock genes show circadian transcriptional oscillations that are served by positive and negative regulatory factors. CLOCK and BMAL are transcription factors constituting a positive regulatory complex that binds to the CACGTG-type E-box, a core circadian enhancer element [1]. Cryptochromes (CRYs) are unique molecules in that they retain the structure of blue-light photoreceptors, which are highly related to photolyases [2], [3], and the vertebrate CRYs operate as negative factors inhibiting E-box-mediated circadian gene transcription [4].

Since the end of the last century, circadian clock components and the circadian clock mechanism have been extensively studied in both vertebrates and invertebrates. However, the studies have mainly concentrated on limited species of mice and fruitflies. Although there are a certain number of reports on clock molecules in zebrafish and *Xenopus laevis* [5], [6], temporal and spatial expression and/or the function of clock molecules in other species, especially in the lower vertebrates, are far less investigated. In this study, we identified two CRYs in the African clawed frog, *Xenopus tropicalis*, one of the most suitable animal models for genetic manipulations in vertebrates. Further investigation into the expression and circadian function of XtCRYs yielded findings of extremely high expression of cryptochrome mRNA in the ovary of this lower vertebrate.

## Results

Based on sequence information from the Ensembl *X. tropicalis* and EST databases, we cloned full-length cDNAs for *XtBmal1*, *XtCry1*, *XtCry2* and *Xtβ2M* (β2-microglobulin) from the adult frog kidney. The full-length *XtClock* sequence has already been identified in the Entrez Nucleotide database (NCBI), and in this study, we were able to determine full-length *XtCRY1, XtCry2, XtBmal1* and *Xtβ2M* sequences. The amino acid sequences of the *X. tropicalis* clock proteins were similar to those of *Xenopus laevis*, a closely related species of *X. tropicalis*, indicating that the main frame of circadian clockwork is conserved between the two *Xenopus* species.

**Figure 1:**
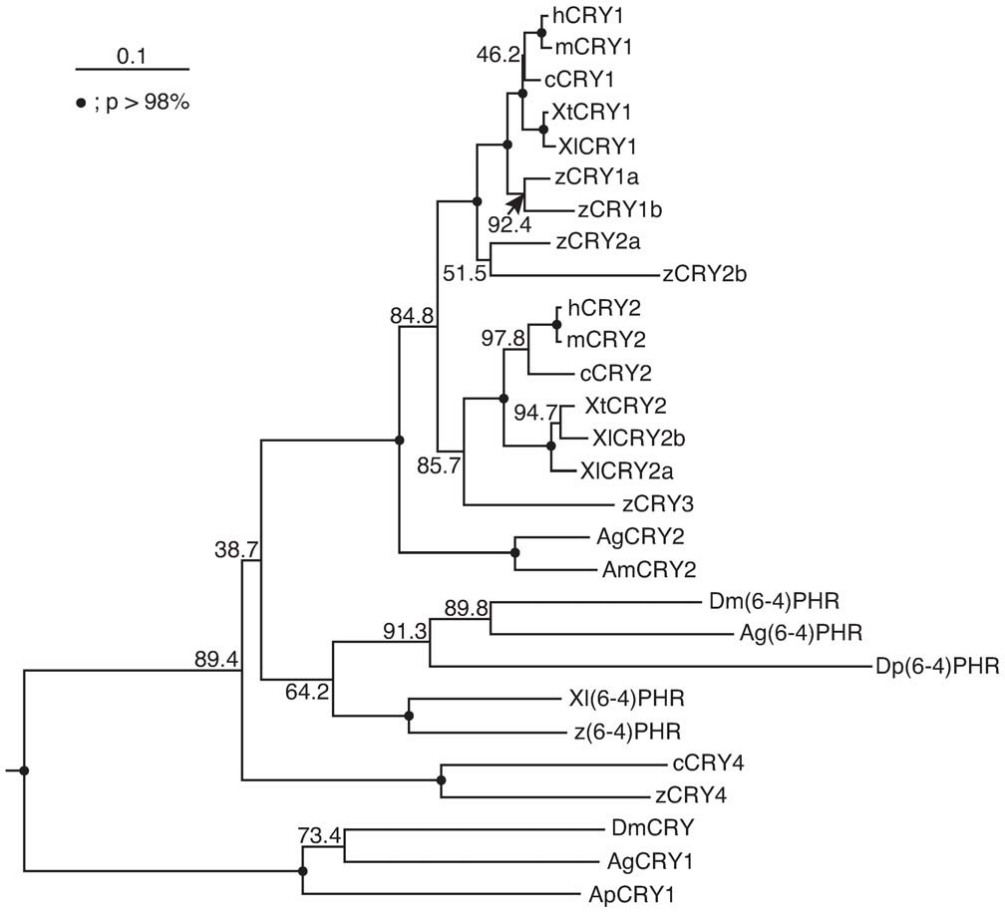
Phylogenetic tree of CRY family proteins. XtCRY sequences and their related sequences obtained from the NCBI Entrez Protein database (accession nos. are shown in Table S1.) were analyzed in the conserved region of the CRY family proteins (corresponding to Arg^10^-Pro^447^ in XtCRY1) using the Neighbor-Joining method and CLUSTAL W. CRY DASH proteins (XlCRY DASH, zCRY DASH, and AtCRY DASH) were used as the outgroup (not shown). Bootstrap probabilities (*p*) are represented by closed circles on the nodes (*p*>98%) or values near the nodes. Abbreviations are defined as follows: h, human; m, mouse; c, chicken; Xl, *Xenopus laevis*; Xt, *Xenopus tropicalis*; z, zebrafish; Ag, *Anopheles gambiae*; Am, *Apis mellifera*; Ap, *Antheraea pernyi*; Dm, *Drosophila melanogaster*; Dp, *Danaus plexippus*; PHR, photolyase.

Deduced amino acid sequences of XtCRYs, XtCLOCK, and XtBMAL1 were aligned with their orthologous protein sequences from other species (Figures S1, S2, and S3) and their evolutionary relationships were analyzed using the Neighbor-Joining (NJ) method (CLUSTAL W version 1.83, http://clustalw.ddbj.nig.ac.jp/) (Figures 1, S4, S5 and Table S1). These NJ trees had nearly identical topologies to those constructed using the maximum-likelihood method in the PHYLIP 3.68 software [7] (data not shown).

**Figure 2:**
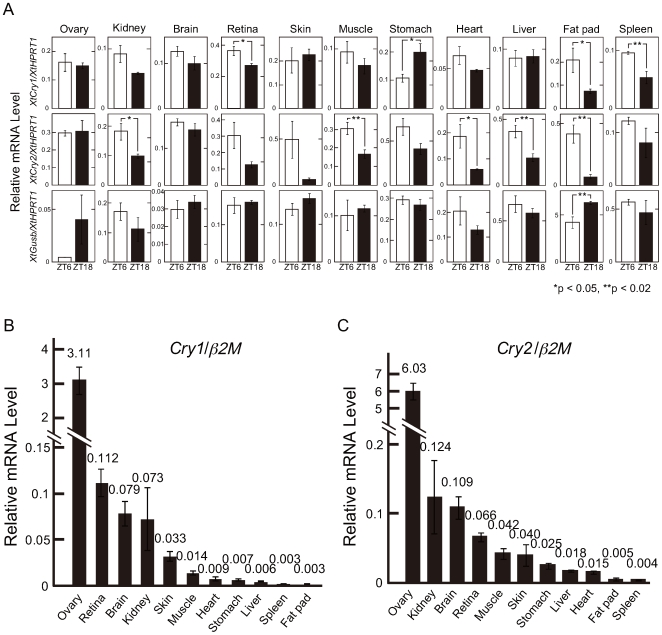
*Cry* mRNA levels and their daily variations in *X. tropicalis* tissues estimated by quantitative RT-PCR. Each tissue (n = 4) was collected at ZT6 and ZT18. Each *Cry* mRNA level was calculated as a value relative to that of the *Xtβ2M* or *XtHprt1* gene. Error bars represent ±SEM. (A) Daily changes in the *Cry* mRNA levels in eleven tissues. Messenger RNA levels are shown as a ratio to *XtHprt1* mRNA levels, which showed relatively small changes between ZT6 and ZT18 in many tissues (except for the ovary). The *Gusb* gene was used as another internal control gene. *p<0.05, ** *p*<0.02, Student's t-test. (B,C) Tissue specificity of *XtCry* mRNA levels. Messenger RNA levels at ZT6 and ZT18 are averaged and shown as a ratio to Xt(2M mRNA levels, which showed relatively small changes among eleven tissues. B; XtCry1, C; XtCry2.

To uncover whether the mRNA levels of *XtCry*s varied in a diurnal or nocturnal manner, we performed quantitative RT-PCR using the cDNA collected from various tissues at midday (ZT6) or midnight (ZT18). In order to establish a consistently transcribed gene for reference, we measured the mRNA levels of three genes, *β2M*, *Hprt1* (hypoxanthine-guanine phosphoribosyltransferase1, Genbank accession no. NM_203981) and *Gusb* (β-glucronidase, Genbank accession no. CT030620). *β2M* and *Hprt1* were selected as the reference control genes for the examination of tissue distribution and diurnal variation, respectively, because the threshold cycle (Ct) values for *β2M* and *Hprt1* remained relatively unchanged for tissues and sampling time, respectively (data not shown). *XtCry2* mRNA levels were significantly higher at midday than at midnight in the kidney, muscle, heart, liver, and fat-pad tissues (Figure 2A). Similar variation was seen in other tissues such as the skin, retina, and stomach. We detected significant variation in *XtCry1* mRNA levels in the spleen, retina, stomach, and fat pads. These changes of *Cry* mRNA expression levels (Figure 2A) are consistent with a possible circadian function of *XtCrys*, but these results do not necessarily indicate that there are endogenous *XtCry* mRNA rhythms, because daily changes in mRNA may be simply driven by light directly or indirectly. Another control gene, *XtGusb,* showed possible nocturnal change in the ovary under these conditions, although this finding was not statistically significant. When the mRNA levels of the two time points (ZT6 and ZT18) were averaged to compare mRNA expression levels among the tissues, the *XtCry* mRNA levels were by far the highest in the ovary versus the other tissues examined (Figure 2B, C). Levels in the ovary were about 28-fold (*XtCry1*) and 48-fold (*XtCry2*) higher than levels in the next abundant tissues, the retina and kidney, respectively (Figure 2B, C).

**Figure 3:**
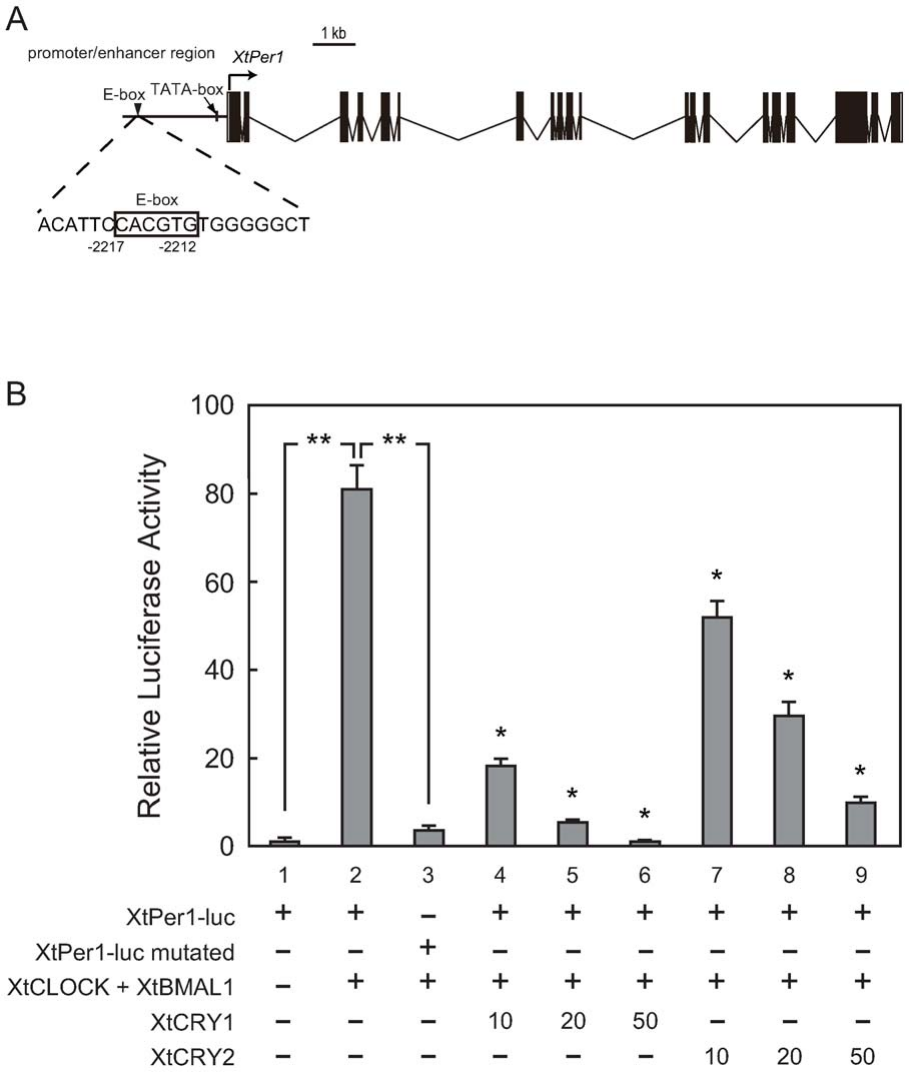
Effects of XtCRY on CLOCK:BMAL1-mediated transcriptional activation from *XtPer1* E-box elements. (A) Schematic diagrams of the *XtPer1* promoter region in which there is one CACGTG E-box (depicted as a closed triangle) and a TATA-box (depicted as a closed box). (B) Twenty-five nanogram of a firefly luciferase reporter with *XtPer1 E-box-SV40-luc* reporter, 0.5 ng of *Renilla* luciferase reporter, pRL-CMV as an internal control (Promega), 125 ng of XtCLOCK expression vector, 12.5 ng of XtBMAL1 expression vector, and 0, 10, 20, or 50 ng of XtCRY expression vector were mixed. The total amount of plasmids was adjusted to 1 µg per well by adding pcDNA3.2/V5-DEST empty vector. All data presented are the means ±SD for three independent experiments. **p<0.0001, Student's t-test, *p<0.05, Tukey-Kramer test, comparing the effect of 4, 5, 6 to 2; or 7, 8, 9 to 2.

**Figure 4:**
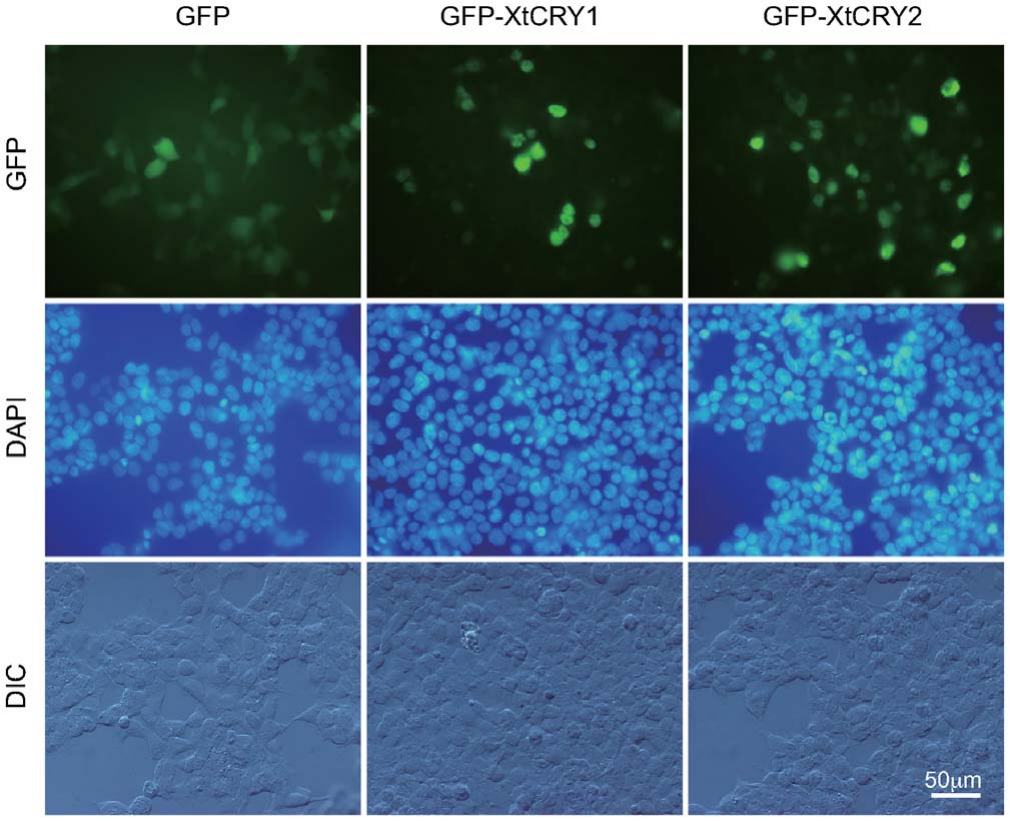
Cellular localization of GFP-fused XtCRYs in HEK 293 cells. Each expression vector was transfected and observed using a fluorescence microscope (upper, GFP; middle, DAPI) or differential interference microscope (lower, DIC).

Next, we performed transcriptional analysis to evaluate whether the putative *X. tropicalis* clock proteins could constitute a circadian molecular loop. At first, the CACGTG-type E-box element and its related sequences in the promoter region of *XtPer1* were examined as a plausible core negative regulator in the circadian molecular loop [8]. Since a CACGTG sequence was found in the −2217∼−2212 upstream region of *XtPer1* (Figure 3A), a tandem repeat of the 20 bp sequence containing the E-box sequence (corresponding to −2224∼−2205 region) was used in the luciferase reporter analysis. Coexpression of XtCLOCK and XtBMAL1 showed more than an 81-fold transactivation from the E-box-containing sequence, and the transactivation was strongly inhibited by XtCRY1 or XtCRY2 in a dose-dependent manner (Figure 3B). Based on reports that nuclear translocation is essential for the E-box-mediated transcription inhibition of CRYs [9], we investigated possible nuclear localization of the XtCRYs by expressing their GFP-fusion proteins in HEK 293 cells. Both GFP-CRY1 and GFP-CRY2 localized in the nucleus, while GFP alone localized predominantly in the cytoplasm (Figure 4).

## Discussion

In this study, we identified two cryptochrome genes in *X. tropicalis* and investigated their mRNA expression in various tissues (Figure 2). Based on the daily variations of both *Cry* mRNAs found in several tissues (Figure 2A), together with their potential function in circadian transcriptional regulation (Figure 3), both CRYs may play a key role in the *X. tropicalis* circadian clock. A comparison of deduced amino acid sequences of the CRYs in the two *Xenopus* species (Tables S2 and S3) indicated that CRY1 is more highly conserved in the two species (97.4%) than CRY2 (92.8%), implying a slower evolution of *Cry1* genes than *Cry2* genes. This implication is also supported by a phylogenetic tree of CRY/Photolyase family proteins (Figure 1) in which vertebrate CRY1 proteins (frog, chicken, and mammalian CRY1) were more closely clustered in comparison with CRY2 proteins.

There is only one *Cry2* gene in *X. tropicalis*, whereas there are two *Cry2* genes in *X. laevis.* In the NJ tree (Figure 1), *XtCry2* directly clustered with *XlCry2b*, and *XlCry2a* positioned itself outside these three *Xenopus Cry2* genes. Therefore, it is likely that duplication of the ancestral *Cry* gene occurred before divergence of the two *Xenopus* species, although we did not find any *Cry2a*-like sequences in the *X. tropicalis* genomic or EST sequences. Alternatively, duplication of the ancestral *XlCry* gene occurred with a genome duplication of ancestral *X. laevis*. This speculation is apparently inconsistent with the local tree topology but agrees well with the fact that extant *X. tropicalis* and *X. laevis* are diploid and pseudo-quadpolyploid, respectively. Further analyses of the genome sequences of both *Xenopus* species, including the genomic location, structure, and organization of *Cry* genes, which are yet unavailable in public databases, can help to expand upon these speculations.

In this study, both *XtCry1* and *XtCry2* transcripts were abundantly expressed in the *X. tropicalis* ovary (Figure 2B, C). There are currently few studies on circadian clock gene expression in the ovary of vertebrates, except for mice and zebrafish (see below), and to the best of our knowledge, the present result is the first report on extremely high accumulation of *Cry* mRNA in the ovary of vertebrates including mice and zebrafish. In mice, *mClock, mBmal1*, and *mCry1* mRNA have been transcribed in the oocyte before fertilization [10], [11], and *mCry1* transcript level was found to be reduced to very low levels until the end of the 1-cell stage after fertilization, while both *mClock* and *mBmal1* transcript levels were not reduced until the end of the 2-cell stage [11]. Presumably, the reduction of *mCry1* transcripts resulted in the relative elevation of *mClock* and *mBmal1* transcripts, and hence transcriptional activation by the CLOCK:BMAL heterodimer would trigger circadian clock-dependent regulation of the cell cycle [12] and/or synchronization of the intrinsic developmental clock of mouse oocytes. Similarly, large amounts of *XtCry1* and *XtCry2* transcripts accumulating in the *Xenopus* ovary might arrest the cell cycle by inhibiting the E-box-controlled cell cycle gene(s). The function of CRY in the ovary is still unknown and will be interesting to be pursued in the future.

In zebrafish, oscillation of *Per3* mRNA in the unfertilized oocyte is reported under light-dark (14:10 h) conditions [13], while the expressions of other clock genes are not reported. This *zPer3* oscillation may indicate that unfertilized zebrafish oocytes have either the presence of an oscillating circadian clock or the capability of using photoreception as a transcriptional control. Mammalian CRY proteins operate as non-photoreceptive transcriptional repressors, whereas avian and invertebrate CRY are likely to retain both photosensitivity and transcriptional regulatory functions [14]–[16]. Although the present study verified only the transcriptional regulatory function of XtCRY1 and XtCRY2 (Figure 3), it might be possible to hypothesize that these CRYs might serve as photoreceptors in the oocyte and control the cirdcadian clock, cell cycle, or hormone secretion according to environmental light conditions.

Recently, it has been suggested that CRYs operate as magnetoreceptors and use light energy for their molecular function [17]. In fact, dCRY, the blue-light circadian photoreceptor in *Drosophila*, is required for light-dependent magnetic response [18]. Because magnetoreception has also been reported in the larvae of *X. laevis* [19], it is important to further characterize XtCRYs by evaluating not only their photoreceptive function but also their possible magnetoreceptive function.

Although amphibian clock genes have been studied in *X. laevis* [6], [8], [20], the present identification and initial characterization of clock genes, including *Cry*s in *X. tropicalis*, is of relative significance. In spite of the close kinship between *X. tropicalis* and *X. laevis*, the following make *X. tropicalis* an advantageous experimental animal model: (i) *X. tropicalis* is a diploid and the genome size is 1.7 Gb, which is smaller than an allotetraploid *X. laevis* (3.1 Gb), (ii) the generation time of *X. tropicalis* (3–4 months) is shorter than that of *X. laevis* (8–12 months) [21], [22], and (iii) *X. tropicalis* is suitable for large-scale breeding because of its smaller body size compared to *X. laevis*. These advantages will encourage further analyses of CRYs that will in turn contribute to a wide variety of research in fields such as reproductive biology, circadian biology, photobiology and magnetochemical biology.

## Materials and Methods

### Animals

Animals were treated in accordance with the guidelines of WASEDA university. Adult *X. tropicalis* were entrained in 12 hr light/dark (LD 12:12 h) cycles for at least two weeks. Tissues were collected at Zeitgeber time 6 (ZT6) under fluorescent light (∼300 µW/cm^2^) or at ZT18 under dim red lighting (>640 nm; ∼120 µW/cm^2^), respectively, and kept in RNA*later* (Ambion) at 4°C until RNA extraction. *X. tropicalis* were kindly provided by Dr. Takase and Dr. Yaoita (the National Bio-Resource Project (NBRP) of the MEXT, Japan; Institute for Amphibian Biology, Graduate school of Science, Hiroshima University), or bred from these frogs.

### Quantitative RT-PCR Analysis

Total RNA was extracted from the tissues using TRIzol reagent (Invitrogen). Quantitative RT-PCR analyses of 1 µg of total RNA were performed using StepOnePlus (Applied Biosystems) along with the High Capacity cDNA Reverse Transcription Kit (Applied Biosystems). The primers for quantitative RT-PCR are described in Table 1.

**Table 1:** List of PCR primers.

Gene	Primer	Sequences
for quantitative RT-PCR		
*XtCry1*	XtCry1-taq-F:	5′-TGGCGTGCTTCCTCACCA-3′
	XtCry1-taq-R:	5′-CCTGCATTCACGCTCCAATCA-3′
*XtCry2*	XtCry2-taq-F:	5′-TCATTATGAAGCTGGCGAAAGAAGC-3′
	XtCry2-taq-R:	5′-CTATGTCCGTTCAGCTCGATTATC-3′
*Xtb2M*	XtB2M-taq-F:	5′-GTGCACATCGACAGCGATG-3′
	XtB2M-taq-R:	5′-GTCTGCGGCTCAGAACATG-3′
*XtGusb*	Xgus-taq-F:	5′-CATGGTGTCAACAAACATGAGGAC-3′
	Xgus-taq-R:	5′-GAGTTAGCACCAAGCCACTTC-3′
*XtHprt1*	Xhprt-taq-F:	5′-AGGCTCAGACATGGCGAG-3′
	Xhprt-taq-R:	5′-GTGGAATGTAGACTTTCTCCAGATC-3′
for cloning and expression vector construction		
*XtClock*	XtClock-Fconst:	5′-CACCGACCTGCCCACCATGAGCTCCACTGCAGACAG-3′
	XtClock-Rconst:	5′-CGTCTCTACTGTTGCTGCACCTTGG-3′
*XtBmal1*	XtBmal1-Fconst:	5′-CACCGACCTGCCCACCATGGCCGACCAAAGAATGG-3′
	XtBmal1-Rconst:	5′-CGTCTCTCACAAAGGCCAAGGTAAGTC-3′
*XtCry1*	XtCry1-Fconst:	5′-CACCGACCTGCCCACCATGGGGGTGAATGCTGTGCAC-3′
	XtCry1-Rconst:	5′-CGTCTCTCAATGGCTGCTTTGCCGTTGG-3′
*XtCry2*	XtCry2-Fconst:	5′-CACCGACCTGCCCACCATGGAGGGGAGACCCTCG-3′
	XtCry2-Rconst:	5′-CGTCTCTCAGAAGTCTTTTGCCGGCCTC-3′

### Statistical Analysis

Data were analyzed using the Student's t-test or ANOVA with Tukey-Kramer multiple comparisons in the Statcel2 (the add-in forms on Excel (Microsoft)) software.

### Cloning of cDNAs of Clock Genes Encoding Full-Length Coding Sequences

Total RNA was extracted from the kidney using TRIzol reagent (Invitrogen). Equal amounts of total RNA from the ZT6 and ZT18 samples were mixed and first-strand cDNAs were synthesized with SuperScript III reverse transcriptase (Invitrogen) using oligo(dT)_21_ primer. Primers for cDNA cloning were designed based on database-deposited partial sequences, which anneal to the untranslated region of *XtClock*, *XtBmal1*, *XtCry1*, *XtCry2* and *Xtβ2M* genes (Table 1) or inside the ORF region of *XtHprt1* and *XrGusb* genes. PCR products amplified with *PfuUltra* (Stratagene) were inserted into the pENTR/D-TOPO vector (Invitrogen), and the inserts of at least three independent clones for each clock gene were sequenced. Full-length cDNAs without presumed PCR errors were isolated.

### Construction of Expression Vectors and *XtPer1 E-box*-*luc* Reporter Vectors

Clock gene expression plasmids were constructed by transferring their cDNA to the pcDNA3.2/V5-DEST vector (Invitrogen) using LR clonase (Invitrogen). To construct the control expression vector without cDNA (pcDNA3.2-empty vector), two oligomers (5′-CACCG ACCTG CCCAC CTGA-3′ and 5′-TCAGG TGGGC AGGTC GGTG-3′) were annealed and inserted into the pENTR/D-TOPO vector to yield the pENTR/D-TOPO-empty vector, which was then treated with the pcDNA3.2/V5-DEST vector and LR clonase (Invitrogen). *XtPer1 E-box-SV40-luc* reporter was constructed using the tandemly-linked CACGTG E-box sequence and its flanking sequence that is found in the *XtPer1* promoter/enhancer region (see below). *XtPer1 mutated-E-box-SV40-luc* reporter was constructed by changing CACGTG to GGACCT. Equal amounts of two oligomers (for *XtPer1 E-box-SV40-luc*, 5′-GATCT ACATTC CACGT GTGGG GGCTA CATTC CACGT GTGGG GGCTA CATTC CACGT GTGGG GGC-3′ and 5′-GATCG CCCCC ACACG TGGAA TGTAG CCCCC ACACG TGGAA TGTAG CCCCC ACACG TGGAA TGTA-3′; for *XtPer1 mutated-E-box-SV40-luc*, 5′-GATCT ACATT CGGAC CTTGG GGGCT ACATT CGGAC CTTGG GGGCT ACATT CGGAC CTTGG GGGC-3′ and 5′- GATCG CCCCC AAGGT CCGAA TGTAG CCCCC AAGGT CCGAA TGTAG CCCCC AAGGT CCGAA TGTA-3′) were annealed and inserted into the *Bgl*II site of the pGL3 promoter vector (Promega).

### Transcriptional Assay

HEK 293 cells (RIKEN CELL BANK) were plated 3.0×10^5^ cells per well on 6-well plates. Plasmid constructs were mixed and transfected into HEK 293 cells using Plus Reagent (Invitrogen) and Lipofectamine Reagent (Invitrogen) according to the manufacturer's instructions. Twenty-four hours after transfection, cell lysates were extracted and transcriptional assays were performed using the Dual-Luciferase assay Kit (Promega).

### Localization of CRY Proteins in HEK 293 Cells

Expression vectors for N-terminally GFP-tagged CRY proteins were constructed by transferring *Cry* cDNA into the pcDNA-DEST53 vector (Invitrogen) using the Gateway system (Invitrogen). The expression vector for GFP alone was constructed by the recombination of pENTR/D-TOPO-empty with pcDNA-DEST53.

HEK 293 cells were plated onto CHAMBER SLIDE (IWAKI). The expression plasmids were transfected into HEK 293 cells using Plus Reagent (Invitrogen) and Lipofectamine Reagent (Invitrogen, described above). Twenty-four hours after transfection, the cells were fixed with 4% paraformaldehyde in PBS (10 mM NaH_2_PO_4_, 140 mM NaCl_2_, 1 mM MgCl_2_, pH 7.4) for 10 min, and then washed with PBS. VECTASHIELD Mounting Medium with DAPI (VECTOR) was dropped onto cells for microscopy.

## Supporting Information

Table S1 Accession nos. of amino acid sequences used for phylogenetic analysis.
(0.04 MB DOC)

Table S2 Homologies among CRY and its related proteins (%).
(0.03 MB XLS)

Table S3 Homologies of clock proteins between *X. tropicalis* and other vertebrates (%).
(0.43 MB EPS)

Figure S1 Alignment of XtCRY proteins and representative members of CRY family proteins. These amino acid sequences were aligned using CLUSTAL W. The symbols depicted in the consensus line were as follows: alphabet, identical amino acid; colon, highly conserved amino acid; single dot, weakly conserved amino acid. Abbreviations are defined as follows: h, human; m, mouse; c, chicken; Xt, *Xenopus tropicalis*; Xl, *Xenopus laevis*; z, zebrafish; Am, *Apis mellifera*; Ag, *Anopheles gambiae*; d or Dm, *Drosophila melanogaster*; Dp, *Danaus plexippus*; At, *Arabidopsis thaliana*.
(0.56 MB EPS)

Figure S2 Alignment of XtCLOCK proteins and representative members of CLOCK family proteins. These amino acids sequences were aligned using CLUSTAL W. The symbols depicted in the consensus line were as follows: alphabet, identical amino acid; colon, highly conserved amino acid; single dot, weakly conserved amino acid. Accession nos. of sequences used for the analysis were as follows: hCLOCK, AAH41878; mCLOCK, NP_031741; cCLOCK, NP_989505; XtCLOCK, NP_001122127; XlCLOCK, AAF34772; zCLOCK, NP_571032; zCLOCK3, NP_840080.
(0.47 MB EPS)

Figure S3 Alignment of XtBMAL1 proteins and representative members of BMAL family proteins. These amino acids sequences were aligned using CLUSTAL W. The symbols depicted in the consensus line were as follows: alphabet, identical amino acid; colon, highly conserved amino acid; single dot, weakly conserved amino acid. Accession nos. of sequences used for the analysis were as follows: hBMAL1b, BAA19935; mBMAL1, NP_031515; cBMAL1, NP_001001463; XtBMAL1, AB534556; XlBMAL1, AAW80970; zBMAL1, NP_571652; hBMAL2a, NP_064568; mBMAL2a, AY005163; cBMAL2, AAL98707; zBMAL2, NP_571653.
(0.49 MB EPS)

Figure S4 Phylogenetic tree of CLOCK proteins. CLOCK sequences and their related sequences obtained from the NCBI Entrez Protein database (accession nos. are described in the Figure S2 legend) were analyzed using the Neighbor-Joining method and CLUSTAL W. hNPAS2 (Genbank accession no. NP_002509) and mNPAS2 (Genbank accession no. NP_032745) were used as outgroups (not shown). Bootstrap probabilities (p) are represented by closed circles on the nodes (p = 100%) or values near the nodes.
(0.40 MB EPS)

Figure S5 Phylogenetic tree of BMAL proteins. BMAL sequences and their related sequences were obtained from the NCBI Entrez Protein database (accession nos. are described in the Figure S3 legend) and analyzed using the Neighbor-Joining method and CLUSTAL W. mARNT (Genbank accession no. NP_001032826) and mARNT2 (Genbank accession no. BC054546) were used as outgroups (not shown). Bootstrap probabilities (p) are represented by closed circles on the nodes (p = 100%) or values near the nodes.
(0.40 MB EPS)
