# Supplementary material for: Cryptochrome Genes Are Highly Expressed in the Ovary of the African Clawed Frog, Xenopus tropicalis
Source: PLoS One. 2010 Feb 17;5(2):e9273. doi: 10.1371/journal.pone.0009273 (PMC2822860; doi:10.1371/journal.pone.0009273)
Supplement: Table S1 — Accession nos. of amino acid sequences used for phylogenetic analysis. (0.04 MB DOC) [file pone.0009273.s001.doc]

| **Table S1.** |  |
| --- | --- |
| **Accession nos. of amino acid sequences used for phylogenetic analysis.** | |
|  |  |
| Protein | Accession No. |
| hCRY1 | NP_004066 |
| mCRY1 | NP_031797 |
| cCRY1 | NP_989576 |
| XlCRY1 | AAK94665 |
| XtCRY1 | AB534557 |
| zCRY1a | NP_571864 |
| zCRY1b | NP_571865 |
| zCRY2a | NP_571866 |
| zCRY2b | NP_571867 |
| XtCRY2 | AB534558 |
| hCRY2 | NP_066940 |
| mCRY2 | NP_034093 |
| cCRY2 | NP_989575 |
| XlCRY2a | NP_001082139 |
| XlCRY2b | NP_001083936 |
| zCRY3 | NP_571861 |
| cCRY4 | AY300013 |
| zCRY4 | NP_571862 |
| Xl(6-4)PHR | NP_001081421 |
| z(6-4)PHR | NP_571863 |
| Ag(6-4)PHR | XP_314748 |
| Dm(6-4)PHR | BAA12067 |
| Dp(6-4)PHR | ABO38436 |
| AgCRY1 | XP_321104 |
| ApCRY1 | AAK11644 |
| dCRY | NP_732407 |
| AgCRY2 | XP_313179 |
| AmCRY2 | NP_001077099 |
| XlCRY DASH | NP_001084438 |
| zCRY DASH | NP 991249 |
| AtCRY DASH | Q84KJ5 |
